# Suboptimal Response to Tenofovir Alafenamide in Two Patients With HBeAg-Positive Hepatitis B: A Case Report

**DOI:** 10.3389/fmed.2021.701061

**Published:** 2021-07-08

**Authors:** Ruochan Chen, Siya Pei, Yayu Chen, Linxia Tan, Ying Xue, Shao Liu, Yan Huang, Xuegong Fan

**Affiliations:** ^1^Department of Infectious Disease, Xiangya Hospital, Central South University, Changsha, China; ^2^Hunan Key Laboratory of Viral Hepatitis, Xiangya Hospital, Central South University, Changsha, China; ^3^Department of Pharmacy, Xiangya Hospital, Central South University, Changsha, China; ^4^The Hunan Institute of Pharmacy Practice and Clinical Research, Changsha, China; ^5^National Clinical Research Center for Geriatric Disorders, Xiangya Hospital, Institute for Rational and Safe Medication Practices, Central South University, Changsha, China

**Keywords:** tenofovir alafenamide, suboptimal response, HBeAg-positive chronic hepatitis B, monotherapy, case report

## Abstract

Tenofovir alafenamide (TAF) is one of the most potent first-line nucleot(s)ide analogs for treating chronic hepatitis B virus (HBV) infections. To date, no cases of TAF drug resistance and/or suboptimal response have been reported. To our knowledge, this is the first report of two adult male patients presenting a suboptimal response response to TAF monotherapy. Our study indicates long-term observations and extensive data are needed to further evaluate the efficacy and safety of TAF, and highlights the need for the development of robust novel direct-acting antivirals and immune therapies for HBV.

## Introduction

Chronic infection with the hepatitis B virus (HBV) occurs in nearly 250 million people globally and more than 80 million people in China (1, 2). Chronic hepatitis B (CHB) infection causes excessive or persistent inflammation in liver, which can lead to adverse clinical outcome like liver fibrosis and cirrhosis, liver decompensation, and even hepatocellular carcinoma. Entecavir (ETV), tenofovir disoproxil fumarate (TDF), and tenofovir alafenamide (TAF) are currently recommended first-line treatmentsfor CHB in international guidelines owing to their high potency and low resistance by the virus (3, 4). Currently in China, ETV and TDF are the most commonly used drugs to treat CHB. However, several problems are increasingly emerging with long-term antiviral therapy. First, drug resistance is gradually increasing, including resistance to ETV and TDF (5–10). Second, a diminished estimated glomerular filtration rate, hypophosphatemia, hyperphosphaturia, and Fanconi syndrome have been reported in patients using TDF (11–13). Lastly, more than 50% of patients infected with CHB are between the ages of 40 and 59 years putting them at a high risk of bone and kidney injury (14).

TAF, a new prodrug of tenofovir similar to TDF, has been recently developed to improve the renal- and bone-safety profile compared to that of TDF, while maintaining similar virological efficacy and safety (15, 16). In addition, studies have shown that the decline of renal injury and bone mineral density induced by the long-term use of TDF may be reversible after switching to TAF (17, 18). TAF was approved in the United States and Japan in November 2016, Europe in January 2017, and China in December 2018 for the treatment of patients with CHB. The American Association for the Study of Liver Disease and European Association for the Study of Liver guidelines have placed TAF as the first-line antiviral therapy for HBV (3, 4, 19). To date, no cases of TAF resistance and/or suboptimal response have been reported.

In this report, we describe two patients with hepatitis B e-antigen (HBeAg)-positive CHB showing suboptimal response to TAF. The data presented here are limited to only two individuals; however, as the population of patients receiving TAF treatment increases in China and the world, clinicians need to pay attention to this phenomenon, and further studies are required to gain better insight into the underlying reasons.

## Case Presentations

**Figure 1:**
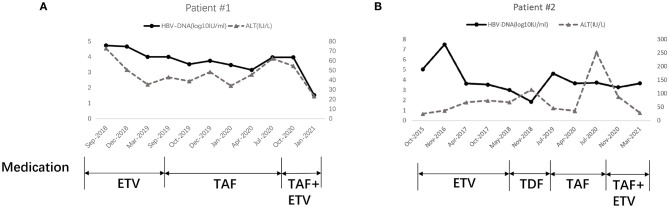
Clinical course of Patient 1# **(A)** and Patient 2# **(B)**. ETV, Entecavir; TDF, Tenofovir disoproxil fumarate; TAF, Tenofovir alafenamide.

A 41-year-old Chinese man (patient #1) with HBeAg-positive CHB (genotype B) was referred to our center in August 2018 because of repeatedly elevated serum alanine aminotransferase (ALT) and total bilirubin (around 37.1–43.5 umol/L). His mother had hepatitis B. He did not have any other family history of hereditary diseases. His AFP level and abdominal ultrasound were normal. His liver elastography was normal (5.1 KPa). Routine blood parameters and the kinetics of HBV-specific antigens and antibodies are shown in Table 1. The detection for HIV and HCV antibody was negative for this patient. He was started on ETV monotherapy (0.5 mg/day) in August 2018, with 1.64 × 10^∧^5 IU/mL of HBV-DNA as the baseline. He continued to take ETV regularly for 12 months, and ALT levels were maintained within the normal range. However, he presented with persistent viremia, with the HBV-DNA level constantly more than 10^∧^4 IU/mL. This patient was treated with TAF (25 mg/day), beginning in September 2019, when his HBV-DNA level was 1 × 10^∧^4 IU/ml. His drug compliance to TAF was assessed in three ways: (i) inquiry by the attending physician at each visit, (ii) medication possession ratio (MPR), which was calculated by the total number of days of medication supply divided by the time interval, and (iii) measurement of serum trough concentrations of TAF using liquid chromatography/mass spectroscopy. Results revealed that his TAF serum concentration was 67 ng/ml 2 h after drug administration; his HBV-DNA was detected at this time. Moreover, the patient confirmed that he had complied with the antiviral regimen, and the MPR exceeded 90%, which indicated good compliance. However, the lowest HBV-DNA level was 3.35 × 10^∧^3 IU/ml during the 12 months of TAF treatment until now. According to the 2017 European Association for the Study of Liver guideline of CHB, 2015 Chinese Prevention and Treatment Guidelines of CHB, and 2015 Asian-Pacific clinical practice guidelines on the management of hepatitis B, this patient met the criteria for primary non-response to TAF (<1 log10 IU/ml decrease in the HBV DNA level from baseline after 3 months of therapy), which suggests a suboptimal response. The reverse transcriptase region of HBV was extracted and amplificated for direct sequencing and clonal analysis. However, no genotypic mutations were detected, including mutations associated with tenofovir resistance (rtA194T, rtS106C, rtH126Y, rtD134E and rtL269I) as well as established ETV-associated mutations (rtM204I/V/L, rtL180M, rtI169T, rtT184A/G/I/S, rtS202G/I, and rtM250V). Other mutations were also detected, such as rtV173L, rtA181T/V, rtQ215S, rtl233V, and rtN236T. The antiviral treatment was changed to a combination of TAF (25 mg/day) and ETV (0.5 mg/day) in October 2020. The clinical course of this patient is shown in Figure 1A.

**Table 1:** Routine blood parameters and the kinetics of HBV-specific antigens and antibodies of patient 1#.

**Date**	**RBC** ** (10^∧^12/L)**	**Hb** ** (g/L)**	**WBC** ** (10^∧^9/L)**	**PLT** ** (10^∧^9/L)**	**Neutrophils** ** (10^**∧**^9/L)**	**Lymphocytes** ** (10^**∧**^9/L)**
**Oct-2019**	**5.10**	**166**	**6.9**	**231**	**4.6**	**1.7**
**Date**	**HBsAg** ** (IU/ml)**	**HBsAb** ** (mIU/ml)**	**HBeAg** ** (S/CO)**	**HBeAb** ** (S/CO)**	**HBcAb** ** (S/CO)**	**HBcAb-IgM** ** (S/CO)**
Dec-2018	32,919.52	0	1324.73	50.72	5.52	0.46
Mar-2019	21,806.19	0	1337.04	48.85	11.47	0.45
Sep-2019	31,041.21	0	202.41	54.50	11.31	0.54
Jan-2020	20,121.08	0	939.44	49.79	11.24	0.49
Mar-2020	16,749.57	0	775.88	45.12	10.12	0.47
Jul-2020	16,944.28	0	976.61	44.49	11.26	0.43
Oct-2020	14,810.62	0	945.66	42.23	11.18	0.39
Jan-2021	11,664.68	0	699.22	34.35	11.12	0.47

*RBC, red blood cell; Hb, hemoglobin; WBC: white blood cell; Plt: platelet; HBsAg: hepatitis B surface antigen; HBsAb: hepatitis B surface antibody; HBeAg: hepatitis B e antigen; HBeAb: hepatitis B e antibody; HBcAb: hepatitis B core antibody*.

Patient #2, a 27-year-old Chinese man with HBeAg-positive CHB (genotype B), was admitted to our center in April 2014. His brother had hepatitis B. He did not have any other family history of hereditary diseases. His AFP level and abdominal ultrasound were normal. His liver elastography was normal (6.0 KPa). Routine blood parameters and the kinetics of HBV-specific antigens and antibodies are shown in Table 2. The detection for HIV and HCV antibody was negative for this patient. He started ETV monotherapy (0.5 mg/day) at that time (Figure 1B). Owing to virus breakthrough, resistance testing in November 2016 (using a similar method as that for patient #1) detected mutant virus populations at positions 180 and 204 (rtL180M, rtM204V/I/L). The antiviral regimen was changed to TDF (300 mg/day). This resulted in his HBV-DNA level decreasing gradually to 70.7 IU/mL in November 2018. The antiviral regimen was changed to TAF (25 mg/day) in July 2019 owing to a virus breakthrough during treatment with 300 mg/day TDF. After 10 months of TAF treatment, the HBV-DNA of this patient was determined to be 4 × 10^∧^3 IU/mL. He also met the criteria for a primary non-response to TAF. This patient complied with his antiviral regimen; an attending physician assessed his compliance at each visit, and the MPR exceeded 90%. The results revealed a TAF serum concentration of 109 ng/ml 2 h after drug administration. The antiviral treatment was changed to a combination of TAF (25 mg/day) and ETV (0.5 mg/day) in April 2020. However, the HBV-DNA and ALT level is even higher after 3 months. Second DNA sequencing detected the same mutant virus populations at positions 180 and 204 (rtL180M, rtM204V/I/L). The antiviral treatment was changed to a combination of TAF (25 mg/day) and ETV (0.5 mg/day) in November 2020. The clinical course of this patient is shown in Figure 1B.

**Table 2:** Routine blood parameters and the kinetics of HBV-specific antigens and antibodies of patient 2#.

**Date**	**RBC** ** (10^**∧**^12/L)**	**Hb** ** (g/L)**	**WBC** ** (10^**∧**^9/L)**	**PLT** ** (10^**∧**^9/L)**	**Neutrophils** ** (10^**∧**^9/L)**	**Lymphocytes** ** (10^**∧**^9/L)**
**Apr-2020**	**5.35**	**157**	**4.4**	**230**	**2.8**	**1.3**
	**HBsAg (IU/mL)**	**HBsAb (mIU/mL)**	**HBeAg (S/CO)**	**HBeAb (S/CO)**	**HBcAg (S/CO)**	**HBcAg-IgM (S/CO)**
May-2018	7,733.96	0	719.62	36.87	11.85	0.28
Nov-2018	2,447.57	0	489.24	22.89	11.69	0.25
Jul-2019	27,160.76	0	1,292.70	60.59	11.24	0.59
Apr-2020	16,454.68	0	983.31	58.67	11.32	0.46
Jul-2020	18,044.41	0	1,155.43	67.56	11.21	0.39
Nov-2020	11,071.80	0	943.58	51.69	11.13	0.47
Mar-2021	5,017.85	0	758.74	39.41	10.48	0.51

*RBC, red blood cell; Hb, hemoglobin; WBC, white blood cell; Plt, platelet; HBsAg, hepatitis B surface antigen; HBsAb, hepatitis B surface antibody; HBeAg, hepatitis B e antigen; HBeAb, hepatitis B e antibody; HBcAb, hepatitis B core antibody*.

## Discussion

According to pharmacokinetics data, TAF reaches a therapeutic concentration in hepatocytes at a lower oral dose (25 mg/day) than does TDF (300 mg/day) (20). Therefore, a small dosage, high distribution in cells, and non-toxicity to bones and kidneys are prominent characteristics of TAF treatment. HBV that displays clinical resistance to TAF has not been reported previously. The current report involves the first two patients with CHB showing a suboptimal response to TAF monotherapy. Baseline HBV DNA levels of these two patients were relatively low (10^∧^4) IU/ml) when they started TAF monotherapy, although both were HBeAg-positive. Initially, we thought the HBV-DNA level should have decreased quickly in these two patients following TAF therapy. However, after at least 10 months of TAF treatment (25 mg/day), the viral load did not decrease as expected. Moreover, their adherence to treatment was ascertained before testing for genotypic resistance; they did not take any other drugs concurrently, which excluded the possibility of a drug interaction. Because most studies regarding the concentration of TAF and its active metabolite in hepatocytes have been conducted in cell and animal models (21, 22), the actual level, stability, and anti-HBV activity of tenofovir in human hepatocytes after administration should be further evaluated.

A major concern with long-term nucleot(s)ide analog treatment is the selection of antiviral-resistant mutations (4). After excluding the possibilities of medication non-adherence and drug interactions, we considered the possibility of HBV genotypic resistance to TAF. TAF shows a higher barrier to drug resistance than lamivudine, adefovir dipivoxil, telbivudine, and even ETV. Currently, phenotypic resistance caused by genotypic resistance to TAF has not been reported (23). No resistance to TAF has been detected via sequence analysis in patients with CHB with a viral breakthrough, an HBV-DNA level ≥ 69 IU/ml at week 24/96, or after TAF withdrawal (24). TAF also demonstrates broad cross-genotype activity against wild-type HBV clinical isolates and is effective against multidrug-resistant HBV isolates *in vitro* (23). Despite high genetic barriers to TFV, emerging evidence has reported that extensive amino acid substitutions were associated with reduced TFV sensitivity, described in both treatment- naïve and -experienced individuals with CHB (6, 8, 10) in Asia, Africa, and Europe. Moreover, in a clinical setting, 0.8–24% patients exhibited a partial response to TDF, while some developed viral breakthrough despite good adherence to TDF (25, 26).

Currently, long-term data on the risk of resistance to TAF and its efficacy are lacking. In our study, both patients underwent testing for genotypic resistance, and the gene encoding HBV reverse transcriptase was sequenced. The HBV genotype was B, and there was no mutation detected in patient #1 according to the results of an analysis in December 2019. Patient #2 was also infected with type B HBV. Mutations of rtL180M and rtM204V were detected in this patient in November 2016. These two mutations are associated with lamivudine, telbivudine, and ETV resistance, both *in vivo* and *in vitro* (27, 28). Theoretically, patient #2 should be sensitive to TDF/TAF monotherapy. However, we do not know whether the resistance profile progressively evolved to a more complex pattern. We sent the sample from patient #1 for gene sequencing in May and July 2020 and no mutation was reported. Although no gene mutation has been characterized, we still suspect the existence of genotypic resistance to TAF in these two patients; however, this needs to be confirmed in a future study.

In summary, we report the first two patients with CHB displaying a suboptimal response to TAF monotherapy in a clinical setting. The most significant limitations of this study are that the underlying cause of TAF non-response remains obscure and the number of cases is limited. However, there are other cases with inferior responses to TAF treatment in our hospital that we wish to document and report in the near future. Nevertheless, based on the current two cases, physicians should pay more attention to patients with CHB who exhibit an unsatisfactory response despite good adherence to tenofovir-containing regimens and try to identify the underlying reasons. Although TAF has been approved as a first-line therapeutic option for CHB in the current international guidelines owing to its high potency and low resistance by the virus, there are still several problems to be addressed. First, although several studies show that TAF has some advantages over TDF, long-term observations and additional data are needed to further evaluate the efficacy and safety of TAF in patients with CHB. Second, an appropriate adjustment to the antiviral regimen for patients who do not obtain a viral response, even after the long-term administration of TAF, is unknown.Increasing the dose or combining with other nucleot(s)ide analogs is one of the approaches. However, even high-dose TAF might not be an optimal rescue therapy for patients who develop tenofovir-resistance, considering that the IC_50_ and IC_90_ values of CYEI mutants are 15.3- and 26.3-fold higher, respectively, than those of the wild-type HBV (6). Furthermore, we should consider the safety profile of a high-dose TAF regimen. Lastly, we should recognize that although all nucleot(s)ide analogs can inhibit HBV replication, they cannot completely eliminate covalently closed circular DNA in hepatocytes. Thus, it is of great significance to develop a curative strategy that enables a functional or even completely sterilizing cure in patients with CHB.

## Data Availability Statement

The original contributions presented in the study are included in the article/Supplementary Material, further inquiries can be directed to the corresponding authors.

## Ethics Statement

Written informed consent was obtained from the individual(s) for the publication of any potentially identifiable images or data included in this article.

## Author Contributions

RC, YH, and XF wrote the manuscript. SP, YC, LT, YX, and SL collected and analyzed the data. All authors read and approved the final manuscript.

## Conflict of Interest

The authors declare that the research was conducted in the absence of any commercial or financial relationships that could be construed as a potential conflict of interest.

## Abbreviations

ALT: alanine aminotransferase
CHB: chronic hepatitis B
ETV: entecavir
HBeAg: hepatitis B e-antigen
HBV: hepatitis B virus
MPR: medication possession ratio
TDF: tenofovirdisoproxil fumarate
TAF: tenofovir alafenamide.

## References

[1] Polaris Observatory Collaborators. Global prevalence, treatment, and prevention of hepatitis B virus infection in 2016: a modelling study. Lancet Gastroenterol Hepatol. (2018) 3:383–403. 10.1016/S2468-1253(18)30056-6 29599078

[2] Schweitzer A Horn J Mikolajczyk RT Krause G Ott JJ. Estimations of worldwide prevalence of chronic hepatitis B virus infection: a systematic review of data published between 1965 and 2013. Lancet. (2015) 386:1546–55. 10.1016/S0140-6736(15)61412-X 26231459

[3] European Association for the Study of the Liver. Electronic address: easloffice@easloffice.eu, European Association for the Study of the Liver. EASL 2017 Clinical Practice Guidelines on the management of hepatitis B virus infection. J Hepatol. (2017) 67:370–98. 10.1016/j.jhep.2017.03.021 28427875

[4] Terrault NA Lok A McMahon BJ Chang KM Hwang JP Jonas MM. Update on prevention, diagnosis, and treatment of chronic hepatitis B: AASLD 2018 hepatitis B guidance. Hepatology. (2018) 67:1560–99. 10.1002/hep.29800 29405329 PMC5975958

[5] Lok AS Zoulim F Locarnini S Bartholomeusz A Ghany MG Pawlotsky JM. Antiviral drug-resistant HBV: standardization of nomenclature and assays and recommendations for management. Hepatology. (2007) 46:254–65. 10.1002/hep.21698 17596850

[6] Park ES Lee AR Kim DH Lee JH Yoo JJ Ahn SH. Identification of a quadruple mutation that confers tenofovir resistance in chronic hepatitis B patients. J Hepatol. (2019) 70:1093–102. 10.1016/j.jhep.2019.02.006 30794889

[7] Baldick CJ Tenney DJ Mazzucco CE Eggers BJ Rose RE Pokornowski KA. Comprehensive evaluation of hepatitis B virus reverse transcriptase substitutions associated with entecavir resistance. Hepatology. (2008) 47:1473–82. 10.1002/hep.22211 18435459

[8] Mokaya J Maponga TG McNaughton AL Van Schalkwyk M Hugo S Singer JB. Evidence of tenofovir resistance in chronic hepatitis B virus (HBV) infection: An observational case series of South African adults. J Clin Virol. (2020) 129:104548. 10.1016/j.jcv.2020.104548 32663786 PMC7408481

[9] Jiang D Wang J Zhao X Li Y Zhang Q Song C. Entecavir resistance mutations rtL180M/T184L/M204V combined with rtA200V lead to tenofovir resistance. Liver Int. (2020) 40:83–91. 10.1111/liv.14241 31498528

[10] Cho WH Lee HJ Bang KB Kim SB Song IH. Development of tenofovir disoproxil fumarate resistance after complete viral suppression in a patient with treatment-naïve chronic hepatitis B: A case report and review of the literature. World J Gastroenterol. (2018) 24:1919–24. 10.3748/wjg.v24.i17.1919 29740207 PMC5937209

[11] Heathcote EJ Marcellin P Buti M Gane E De Man RA Krastev Z. Three-year efficacy and safety of tenofovir disoproxil fumarate treatment for chronic hepatitis B. Gastroenterology. (2011) 140:132–43. 10.1053/j.gastro.2010.10.011 20955704

[12] Marcellin P Gane E Buti M Afdhal N Sievert W Jacobson IM. Regression of cirrhosis during treatment with tenofovir disoproxil fumarate for chronic hepatitis B: a 5-year open-label follow-up study. Lancet. (2013) 381:468–75. 10.1016/S0140-6736(12)61425-1 23234725

[13] Petersen J Heyne R Mauss S Schlaak J Schiffelholz W Eisenbach C. Effectiveness and safety of tenofovir disoproxil fumarate in chronic hepatitis B: a 3-year prospective field practice study in Germany. Dig Dis Sci. (2016) 61:3061–71. 10.1007/s10620-015-3960-x 26576555

[14] Wu W Zhu Y Yu C Yang S Ruan B Chen Y. Clinical features of treatment-naive patients with hepatitis B virus infection: a community-based survey from high- and intermediate-hepatitis B endemicity regions in Southeast China. Medicine. (2017) 96:e6660. 10.1097/MD.0000000000006660 28422873 PMC5406089

[15] Buti M Gane E Seto WK Chan HL Chuang WL Stepanova T. Tenofovir alafenamide versus tenofovir disoproxil fumarate for the treatment of patients with HBeAg-negative chronic hepatitis B virus infection: a randomised, double-blind, phase 3, non-inferiority trial. Lancet Gastroenterol Hepatol. (2016) 1:196–206. 10.1016/S2468-1253(16)30107-8 28404092

[16] Chan HL Fung S Seto WK Chuang WL Chen CY Kim HJ. Tenofovir alafenamide versus tenofovir disoproxil fumarate for the treatment of HBeAg-positive chronic hepatitis B virus infection: a randomised, double-blind, phase 3, non-inferiority trial. Lancet Gastroenterol Hepatol. (2016) 1:185–95. 10.1016/S2468-1253(16)30024-3 28404091

[17] Seto WK Asahina Y Brown TT Peng CY Stanciu C Abdurakhmanov D. Improved bone safety of tenofovir alafenamide compared to tenofovir disoproxil fumarate over 2 years in patients with chronic HBV infection. Clin Gastroenterol Hepatol. (2018). 10.1016/j.cgh.2018.06.023 29933096

[18] Fong TL Lee BT Tien A Chang M Lim C Ahn A. Improvement of bone mineral density and markers of proximal renal tubular function in chronic hepatitis B patients switched from tenofovir disoproxil fumarate to tenofovir alafenamide. J Viral Hepat. (2019) 26:561–7. 10.1111/jvh.13053 30576085

[19] Tong MJ Pan CQ Han SB Lu DS Raman S Hu KQ. An expert consensus for the management of chronic hepatitis B in Asian Americans. Aliment Pharmacol Ther. (2018) 47:1181–200. 10.1111/apt.14577 29479728 PMC5900913

[20] Ruane PJ DeJesus E Berger D Markowitz M Bredeek UF Callebaut C. Antiviral activity, safety, and pharmacokinetics/pharmacodynamics of tenofovir alafenamide as 10-day monotherapy in HIV-1-positive adults. J Acquir Immune Defic Syndr. (2013) 63:449–55. 10.1097/QAI.0b013e3182965d45 23807155

[21] Murakami E Wang T Park Y Hao J Lepist EI Babusis D. Implications of efficient hepatic delivery by tenofovir alafenamide (GS-7340) for hepatitis B virus therapy. Antimicrob Agents Chemother. (2015) 59:3563–9. 10.1128/AAC.00128-15 25870059 PMC4432166

[22] Babusis D Phan TK Lee WA Watkins WJ Ray AS. Mechanism for effective lymphoid cell and tissue loading following oral administration of nucleotide prodrug GS-7340. Mol Pharm. (2013) 10:459–66. 10.1021/mp3002045 22738467

[23] Liu Y Miller MD Kitrinos KM. Tenofovir alafenamide demonstrates broad cross-genotype activity against wild-type HBV clinical isolates and maintains susceptibility to drug-resistant HBV isolates in vitro. Antiviral Res. (2017) 139:25–31. 10.1016/j.antiviral.2016.12.012 28017761

[24] Cathcart AL Chan HL Bhardwaj N Liu Y Marcellin P Pan CQ. No resistance to tenofovir alafenamide detected through 96 weeks of treatment in patients with chronic hepatitis B infection. Antimicrob Agents Chemother. (2018) 62:e01064–18. 10.1128/AAC.01064-18 30038044 PMC6153810

[25] Marcellin P Heathcote EJ Buti M Gane E de Man RA Krastev Z. Tenofovir disoproxil fumarate versus adefovir dipivoxil for chronic hepatitis B. N Engl J Med. (2008) 359:2442–55. 10.1056/NEJMoa0802878 19052126

[26] Kitrinos KM Corsa A Liu Y Flaherty J Snow-Lampart A Marcellin P. No detectable resistance to tenofovir disoproxil fumarate after 6 years of therapy in patients with chronic hepatitis B. Hepatology. (2014) 59:434–42. 10.1002/hep.26686 23939953

[27] Huang ZB Zhao SS Huang Y Dai XH Zhou RR Yi PP. Comparison of the efficacy of Lamivudine plus adefovir versus entecavir in the treatment of Lamivudine-resistant chronic hepatitis B: a systematic review and meta-analysis. Clin Ther. (2013) 35:1997–2006. 10.1016/j.clinthera.2013.10.002 24238791

[28] Kim HJ Park JH Park DI Cho YK Sohn CI Jeon WK. Rescue therapy for lamivudine-resistant chronic hepatitis B: comparison between entecavir 1.0 mg monotherapy, adefovir monotherapy and adefovir add-on lamivudine combination therapy. J Gastroenterol Hepatol. (2010) 25:1374–80. 10.1111/j.1440-1746.2010.06381.x 20659226

